# Association between specialized nutrition support and 90‐day mortality relative to standard of care in malnourished adults with decompensated cirrhosis: A retrospective cohort study

**DOI:** 10.1002/jpen.70066

**Published:** 2026-02-20

**Authors:** Katharina L. Hupa‐Breier, Laura Buttler, Claudia Seipt, Andrea Markowski, Marie Griemsmann, Tammo L. Tergast, Birgit Kaufmann, Hannah Rieland, Heiner Wedemeyer, Benjamin Maasoumy, Andrea Schneider

**Affiliations:** ^1^ Department of Gastroenterology, Hepatology, Infectious Diseases and Endocrinology Hannover Medical School Hannover Germany; ^2^ German Centre for Infection Research (DZIF) partner‐site Hannover‐Braunschweig Hannover Germany; ^3^ Hannover Medical School Excellence Cluster RESIST Hannover Germany

**Keywords:** hepatic encephalopathy, home parenteral nutrition, liver cirrhosis, malnutrition

## Abstract

**Background:**

Malnutrition is common among patients with decompensated liver cirrhosis and linked to poor prognosis. Guidelines recommend intensified nutrition support e.g. parenteral nutrition, but evidence regarding safety and effectiveness is scarce. We aimed to investigate the impact of nutrition support, specifically parenteral nutrition, on mortality (primary end point) and on cirrhosis‐specific complications (secondary end points) in patients with advanced liver cirrhosis.

**Methods:**

Consecutive malnourished patients with decompensated cirrhosis treated at our center between 2013 and 2018 were investigated. Fifty‐three patients received specialized nutrition support; 33 received parenteral nutrition. The specialized nutrition support group (cohort 1) and the home parenteral nutrition group (cohort 2) were compared with patients without specific dietary support (standard‐of‐care group) after 1:1 and 1:2 propensity score matching, respectively. Mortality, hepatic encephalopathy, infections, and rehospitalization were investigated within 90 days.

**Results:**

Median baseline Model for End‐Stage Liver Disease score (possible range 6–40 points) was 18 in cohort 1 and 16 in cohort 2. No impact of specialized nutrition support on the clinical outcome was detected. Between cohort 2 and standard of care, no differences in mortality and rehospitalization were observed. Whereas parenteral nutrition was associated with increased risk for bloodstream infections (hazard ratio [HR] = 21.3; *P* = 0.004), overall incidence of bacterial infections was comparable between groups (HR = 0.82; *P* = 0.45). Of note, the likelihood for hepatic encephalopathy was significantly reduced in cohort 2 in the multivariable competing risk model (HR = 0.28; *P* = 0.03).

**Conclusion:**

Home parenteral nutrition seems safe overall and may ameliorate the risk for hepatic encephalopathy in candidates for liver transplant.

**Abbreviations**
BMI: body mass index
EASL: European Association for the Study of the Liver
ESPEN: European Society for Clinical Nutrition and Metabolism
HR: hazard ratio
MELD: Model for End‐Stage Liver Disease
NRS‐2002: Nutritional Risk Screening 2002

## INTRODUCTION

In a stage of advanced liver cirrhosis, up to 70% of patients are affected by malnutrition.^1^, ^2^ Patients with advanced liver cirrhosis have a significantly increased risk for complications, and this is further aggravated by malnutrition and associated with a poor prognosis.^3^ Particularly in candidates for liver transplant, malnutrition has been associated with an increased waitlist mortality and a higher likelihood for unfavorable outcomes after liver transplant.^2^ Consequently, current guidelines recommend intensified oral nutrition support for patients with decompensated liver cirrhosis, particularly in the pretransplant setting.^4^, ^5^ Given that many patients fail to meet their energy and protein requirements through oral dietary intake, additional enteral or parenteral nutrition support is required.^6^ Enteral strategies include nasogastric or percutaneous endoscopic gastrostomy tube feeding, which have been linked to improved outcomes.^7^ However, these options might be limited in patients with cirrhosis, especially in the presence of ascites or esophageal varices. In this case, guidelines advocate supportive parenteral nutrition.^4^, ^5^ Besides avoiding prolonged starving periods under clinical conditions, home parenteral nutrition allows long‐term continuation of nutrition therapy after hospital discharge.^8^ Nevertheless, far too little attention has been paid to the impact of specialized nutrition support and particular of home parenteral nutrition on the risk for death and cirrhosis‐specific complications. It is not yet clear whether home parenteral nutrition is linked to an effective reduction of mortality and clinical complications in patients with end‐stage cirrhosis, and uncertainty still exists regarding the impact of parenteral nutrition on infectious complications in this vulnerable collective.

In terms of cirrhosis‐related complications, a relationship between poor dietary conditions and hepatic encephalopathy has been identified.^9^, ^10^ In a condition of malnourishment, increased levels of ammonia, typically following the activation of catabolic pathways and accelerated muscle depletion, promote development of hepatic encephalopathy episodes.^11^, ^12^, ^13^ Consequently, the nutrition status of these patients might be an essential therapeutic target to alleviate morbidity and improve survival. However, permanent central venous catheters, which are required for long‐term home parenteral nutrition,^8^, ^14^ might be an entrance route for pathogens, as they represent a direct access to patients’ circulation and are subject to daily manipulation. Thus, many clinicians have concerns about the onset of long‐term parenteral feeding in these highly vulnerable patients. Because of a disrupted intestinal barrier and a dysbalanced microbiome, the end‐stage of liver disease is accompanied by bacterial translocation and influx of pathogen‐associated molecular patterns, contributing to chronic systemic inflammation.^15^ Finally, these factors promote cirrhosis‐associated immune dysfunction, which leads to a highly elevated infection susceptibility.^16^, ^17^, ^18^, ^19^ Bacterial infections in turn aggravate the likelihood of severe cirrhosis‐related complications and have been linked to a high fatality rate.^20^ Even though nutrition support is required in malnourished patients, infection susceptibility might impair the safety of home parenteral nutrition in the fragile state of advanced liver disease.

We hypothesized that specialized nutrition support is associated with decreased 90‐day mortality in malnourished hospitalized adults with decompensated cirrhosis compared with standard of care. Furthermore, we also hypothesized that home parenteral nutrition is associated with an ameliorated 90‐day mortality in malnourished hospitalized adults with decompensated cirrhosis compared with standard of care. The aim of our retrospective cohort study therefore was (1) to examine the effect of specialized nutrition support and home parenteral nutrition on mortality and cirrhosis‐related complications and (2) to analyze the safety of supportive home parenteral nutrition among patients with advanced liver cirrhosis.

## METHODS

### Study cohort

**Figure 1:**
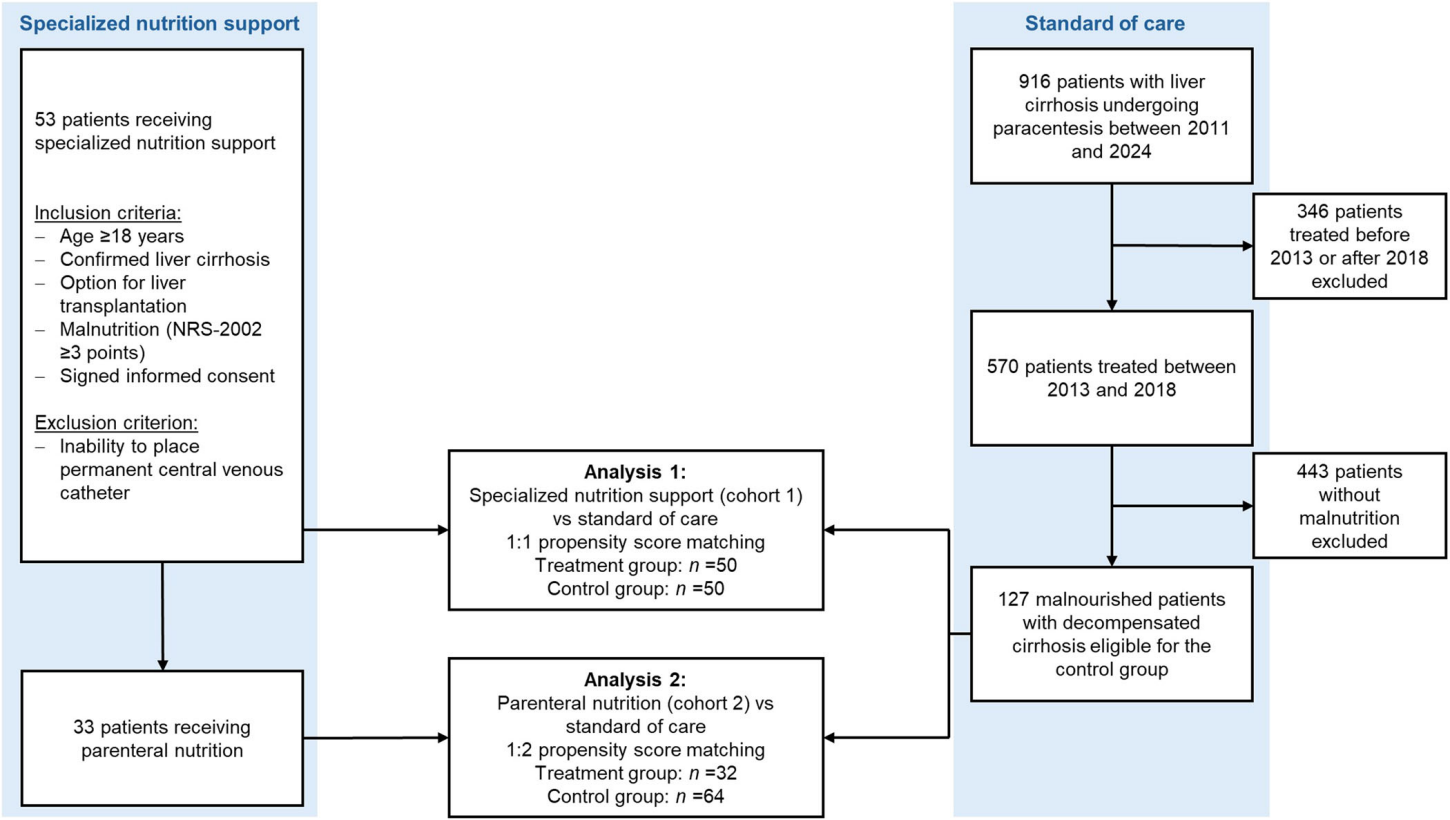
Study cohort and study design. NRS‐2002, Nutritional Risk Screening 2002.

We performed a retrospective cohort study in 180 malnourished adults with decompensated cirrhosis hospitalized at the Hannover Medical School Hospital from 2013 to 2018. The study comprised two cohorts derived from 127 malnourished patients with decompensated cirrhosis receiving standard of care and 53 malnourished patients with decompensated cirrhosis receiving specialized nutrition support of whom 33 received parenteral nutrition support integrative to oral feeding (Figure 1). Two analytical cohorts were identified: (1) specialized nutrition support, referred to as cohort 1 (*n* = 50), vs standard of care (*n* = 50) and (2) home parenteral nutrition, referred to as cohort 2 (*n* = 32), vs standard of care (*n* = 64).

For cohort 1, our exposure of interest was specialized nutrition support, defined as repetitive dietary counseling and/or additional home parenteral nutrition. For cohort 2, our exposure of interest was home parenteral nutrition. Our comparator for both cohort 1 and cohort 2 was standard of care, defined as dietary recommendations for patients with decompensated liver cirrhosis based on the 2019 guidelines of the European Association for the Study of the Liver (EASL) and the European Society for Clinical Nutrition and Metabolism (ESPEN) clinical guidelines.^4^, ^5^ Inclusion criteria were age ≥18 years, confirmed liver cirrhosis with evaluation for liver transplant and malnutrition. Risk of malnutrition was defined as at least 3 points on Nutritional Risk Screening 2002 (NRS‐2002) before study inclusion, followed by a detailed assessment and diagnosis of malnutrition (evaluation of the unintended weight loss, body mass index [BMI], and measurement of body cell mass and phase angle by using bioelectrical impedance analysis). Nutrition risk screening was performed either by the treating physicians or by experienced dietitians. Additionally, handgrip strength was determined using a handgrip dynamometer.

Specialized nutrition support included regular nutrition counseling performed by a qualified nutrition assistant at regular intervals every 4 weeks. The energy target was calculated based on anthropometric data or bioelectrical impedance analysis. Patients’ protein and energy intake was determined by dietary recall. Patients who did not achieve their per‐body‐weight energy and protein goals of 35 kcal/kg and 1.5 g/kg, respectively, through oral nutrition were advised to start home parenteral nutrition.^5^ Home parenteral nutrition was initiated and supervised by an established interdisciplinary nutrition support team and continued at home by trained healthcare professionals following standardized hygienic protocols. Inability to place a permanent central venous catheter was defined as an exclusion criterion.

However, some patients refused home parenteral nutrition. In this case, these patients received repetitive dietary counseling with additive individual oral supplementation within the study. Parenteral nutrition was administered at an infusion rate of 1.8 ml/kg/h, up to a maximum daily dose of 35 ml/kg. Patients with special metabolic needs received home parenteral nutrition with individualized compositions. Table 1 presents the detailed energy and protein intake in the subgroups of SNS. Inability to place a permanent central venous catheter was defined as exclusion criterion. The standard‐of‐care nutrition was defined as a single consultation during which the recommendations of the 2019 EASL guidelines for patients with decompensated liver cirrhosis were explained.^5^

**Table 1:** Specification of baseline characteristics of patients receiving SNS and HPN.

	Cohort 1 (SNS group; *n* = 50)	Cohort 1 subgroup: Only oral nutrition (*n* = 18)	Cohort 2 (HPN group; *n* = 32)
Energy intake, kcal/kg/day	27.1 (18.4–35.5)	21.1 (15.1–26.2)	33.5 (26.6–38.8)
Protein intake, g/kg/day	1.2 (0.6–1.6)	0.7 (0.5–1.0)	1.5 (1.2–1.8)
Body cell mass, kg	17.4 (13.5–23.3)	21.3 (14.1–24.9)	16.5 (13.0–22.3)
Phase angle, °	3.3 (2.7–3.9)	3.7 (3.1–4.4)	3.1 (2.7–3.7)
Handgrip strength, kg	36.8 (26.1–67.9)	56.5 (31.7–88.7)	34.8 (23.8–51.5)

*Note*: Values are median (IQR).

Abbreviations: HPN, home parenteral nutrition; SNS, specialized nutrition support

Cohort 1 and cohort 2 were compared with patients who only received standard‐of‐care nutrition treatment but did not undergo any specific dietary intervention. This standard‐of‐care group includes patients with decompensated liver cirrhosis and clinical diagnosis of malnutrition and/or BMI ≤18.5 kg/$m^{2}$. Standard of care includes only basic dietary recommendations for patients with liver cirrhosis based on the current EASL and ESPEN clinical guidelines.^4^, ^5^ Respecting these criteria, 127 patients were eligible for the control group. In the first approach, we compared cohort 1 (specialized nutrition support) with the standard‐of‐care group. In a second approach, cohort 2 (home parenteral nutrition) was compared with the standard‐of‐care group (Figure 1).

### Study design

First, cohort 1 was matched with the standard‐of‐care group in a 1:1 nearest‐neighbor propensity score matching (caliper 0.029). Three patients of cohort 1 without appropriate matching partner were excluded. Hence, 50 patients of the treatment group were matched with 50 patients of the standard‐of‐care group (Figure S1).

Second, we aimed to analyze the safety and efficacy of home parenteral nutrition. Therefore, cohort 2 was matched with the standard‐of‐care group in a 1:2 nearest‐neighbor propensity score matching (caliper 0.051). One patient without a matching partner was excluded, so 32 patients treated parenterally and 64 patients without dietary support remained for analyses (Figure S2). Matching criteria were Model for End‐Stage Liver Disease score (MELD), age, BMI, and serum sodium. Matching was considered to be successful with absolute standardized mean differences between −0.1 and 0.1 (Figures S1A and S2A). For calculation of BMI, weight without ascites was assessed by using patients’ self‐reported dry weight or body weight, which was documented in medical records before hydropic decompensation or after paracentesis.^5^

Our primary outcome for both cohort 1 and cohort 2 was 90‐day mortality, defined as all‐cause mortality within 90 days of hospital admission and determined by hospital records and publicly available mortality data. Our secondary outcomes for both cohort 1 and cohort 2 were overt hepatic encephalopathy, nonelective hospital readmission, any bacterial infections, and bloodstream infections. All end points were studied within 90 days after study inclusion.

Overt hepatic encephalopathy was defined according to the West Haven criteria (signs of at least one of the following criteria: disorientation, lethargy or apathy, inappropriate or bizarre behavior, confusion, asterixis, and lack of awareness reaching from somnolence to coma).^21^ Readmission was defined as first nonelective rehospitalization after discharge from the baseline hospital stay during which the patients were added to the study. Bacterial infections were defined based on the following criteria^22^:

- Bloodstream infection: positive blood cultures
- Urinary tract infection: leukocyturia and clinical signs of infection
- Spontaneous bacterial peritonitis: at least 250 polymorphonuclear or 500 nucleus‐containing cells per cubic millimeter of ascites fluid
- Pneumonia: pulmonary infiltrates in x‐ray or computed tomography imaging
- Infection with unknown focus: suspected infection without identifiable source.

Analysis of overt hepatic encephalopathy was repeated in a multivariable model, adjusted for rifaximin intake because rifaximin is used for hepatic encephalopathy prophylaxis and because administration differed significantly between groups (Tables 2 and 3). Competing risk analyses were performed using liver transplant as competitor for the primary end point and liver transplant or death as competitor for the secondary end points. Patients were censored at the end of follow‐up. Furthermore, patients of the home parenteral nutrition group were censored when the central venous catheter was explanted, and the controls were censored if home parenteral nutrition was started within the follow‐up.

**Table 2:** Baseline characteristics of patients receiving SNS and those who received SOC treatment after 1:1 propensity score matching.

	Cohort 1 (SNS group; *n* = 50)	Matched SOC group (*n* = 50)	*P* value
Age, years	55.2 (50.1–62.4)	58.7 (50.3–62.8)	0.33^a^
Sex, *n* (%)			0.38^b^
Male	30 (60.0)	25 (50.0)	
Female	20 (40.0)	25 (50.0)	
Etiology, *n* (%)			
ALD	18 (36.0)	27 (54.0)	0.12^b^
MetALD	3 (6.0)	2 (4.0)	1.00^b^
MASLD	3 (6.0)	1 (2.0)	0.63^b^
Viral	5 (10.0)	4 (8.0)	1.00^b^
PSC	3 (6.0)	0 (0.0)	0.25^b^
PBC	5 (10.0)	1 (2.0)	0.22^b^
AIH	2 (4.0)	0 (0.0)	0.50^b^
Cryptogenic	3 (6.0)	8 (16.0)	0.18^b^
Others	9 (18.0)	10 (20.0)	1.00^b^
MELD	18 (13–22)	17 (13–22)	0.63^a^
Potassium, mmol/L	4.2 (3.8–4.6)	4.0 (3.5–4.5)	0.15^a^
Sodium, mmol/L	136.0 (129.5–138.0)	135.0 (130.0–138.0)	0.91^a^
Leukocytes, Tsd/µL	7.6 (4.9–9.9)	8.1 (5.8–12.1)	0.12^a^
Hemoglobin, g/dl	9.6 (8.3–10.7)	9.8 (8.7–11.8)	0.04^a^
Platelets, Tsd/µL	104.5 (61.8–143.8)	145.0 (89.5–235.0)	0.01^a^
INR	1.4 (1.2–1.6)	1.4 (1.3–1.6)	0.44^a^
Bilirubin, µmol/L	35.5 (14.0–70.5)	31.0 (16.8–63.5)	0.10^a^
Creatinine, µmol/L	119.5 (80.0–171.3)	114.5 (72.0–157.0)	0.94^a^
Aspartate aminotransferase, U/L	55.0 (36.8–108.3)	50.5 (34.5–77.8)	0.52^a^
Alanine aminotransferase, U/L	28.0 (21.3–67.3)	26.0 (18.8–41.3)	0.14^a^
Alkaline phosphatase, U/L	163.5 (114.8–256.0)	143.0 (104.3–189.5)	0.11^a^
Gamma‐glutamyltransferase, U/L	106.5 (51.8–212.5)	122.5 (63.8–245.8)	0.59^a^
CRP, mg/L	17.9 (7.8–36.3)	24.6 (13.0–38.2)	0.69^a^
Serum cholinesterase, kU/L	1.5 (1.0–1.9)	2.0 (1.3–2.7)	0.03^a^
Serum albumin level, g/L	25.0 (22.3–28.0)	25.0 (21.0–30.5)	0.22^a^
BMI, kg/m^2^	20.9 (18.3–23.8)	20.2 (17.8–23.3)	0.31^a^
Diabetes mellitus, *n* (%)	15 (30.0)	9 (18.0)	0.24^b^
Co‐medication, *n* (%)			
NSBB	16 (32.0)	13 (26.0)	0.68^b^
Norfloxacin	11 (22.0)	1 (2.0)	0.01^b^
PPI	43 (86.0)	38 (76.0)	0.30^b^
Any hepatic encephalopathy prophylaxis	33 (66.0)	27 (54.0)	0.26^b^
Rifaximin	16 (32.0)	7 (14.0)	0.05^b^
Lactulose	27 (54.0)	25 (50.0)	0.83^b^
Ornithine aspartate	17 (34.0)	9 (18.0)	0.10^b^

*Note*: Categorial values are *n* (%), and continuous variables are median (IQR).

Abbreviations: AIH, autoimmune hepatitis; ALD, alcohol‐related liver disease; BMI, body mass index; CRP, C‐reactive protein; INR, international normalized ratio; MASLD, metabolic dysfunction–associated liver disease; MELD, Model for End‐Stage Liver Disease; MetALD, metabolic dysfunction– and alcohol‐related liver disease; NSBB, nonselective beta‐blocker; PBC, primary biliary cholangitis; PPI, proton pump inhibitors; PSC, primary sclerosing cholangitis; SNS, specialized nutrition support; SOC, standard of care. Tsd, one thousand.

^a^ Wilcoxon test was used for continuous parameters.

^b^ McNemar test was used for comparison of categorical variables.

**Table 3:** Baseline characteristics of patients receiving HPN and those who received SOC treatment after 1:2 propensity score matching.

	Cohort 2 (HPN group; *n* = 32)	Matched SOC group (*n* = 64)	*P* value^a^
Age, years	53.8 (48.0–65.5)	57.9 (46.4–64.2)	0.80^b^
Sex, *n* (%)			
Male	19 (59.4)	40 (62.5)	0.94^a^
Female	13 (40.6)	24 (37.5)	
Etiology, *n* (%)			
ALD	12 (37.5)	34 (53.1)	0.22^a^
MetALD	2 (6.3)	3 (4.7)	0.87^a^
MASLD	3 (9.4)	1 (1.6)	0.21^a^
Viral	4 (12.5)	10 (15.6)	0.92^a^
PSC	2 (6.3)	0 (0.0)	0.21^a^
PBC	1 (3.1)	3 (4.7)	0.86^a^
AIH	0 (0.0)	1 (1.6)	0.72^a^
Cryptogenic	2 (6.3)	5 (7.8)	0.89^a^
Others	6 (18.8)	13 (20.3)	0.93^a^
MELD	16 (16–22)	16 (13–22)	0.80^b^
Potassium, mmol/L	4.3 (3.8–4.9)	4.2 (3.7–4.9)	0.95^b^
Sodium, mmol/L	134.0 (126.0–136.0)	132.0 (127.3, 136.0)	0.58^b^
Leukocytes, Tsd/µL	8.3 (5.1–12.0)	7.5 (5.8, 10.8)	0.86^b^
Hemoglobin, g/dl	9.3 (8.2–10.1)	10.5 (8.8, 12.0)	0.01^b^
Platelets, Tsd/µL	105.0 (65.3–138.0)	140.0 (87.3, 185.0)	0.17^b^
INR	1.4 (1.2–1.6)	1.3 (1.3, 1.5)	0.49^b^
Bilirubin, µmol/L	29.5 (14.0–67.0)	35.5 (17.0, 70.5)	0.90^b^
Creatinine, µmol/L	122.0 (79.3–171.8)	118.0 (84.3, 160.0)	0.42^b^
Aspartate aminotransferase, U/L	60.0 (35.3–102.5)	48.0 (33.0, 78.5)	0.46^b^
Alanine aminotransferase, U/L	30.0 (19.8–58.8)	26.0 (19.0, 43.5)	0.76^b^
Alkaline phosphatase, U/L	161.5 (107.0–268.8)	149.5 (105.8, 190.5)	0.20^b^
Gamma‐glutamyltransferase, U/L	113.5 (65.3–245.8)	123.0 (54.0, 224.8)	0.76^b^
CRP, mg/L	26.4 (12.0–43.0)	22.6 (10.0, 38.0)	0.44^b^
Serum cholinesterase, kU/L	1.4 (0.9–1.8)	2.1 (1.6, 2.9)	<0.01^b^
Serum albumin level, g/L	25.0 (22.0–28.0)	26.0 (22.0, 29.5)	0.59^b^
BMI, kg/m^2^	20.5 (17.6–23.2)	19.5 (17.3, 23.4)	0.99^b^
Diabetes mellitus, *n* (%)	9 (28.1)	10 (15.6)	0.24^a^
Co‐medication, *n* (%)			
NSBB	11 (34.4)	20 (31.3)	0.94^a^
Norfloxacin	10 (31.3)	1 (1.6)	<0.001^a^
PPI	29 (90.6)	50 (78.1)	0.22^a^
Any hepatic encephalopathy prophylaxis	24 (75.0)	31 (48.4)	0.02
Rifaximin	17 (53.1)	9 (14.1)	<0.001
Lactulose	18 (56.3)	28 (43.8)	0.35^a^
Ornithine aspartate	12 (37.5)	11 (17.2)	0.05^a^

*Note*: Categorial values are *n* (%), and continuous variables are median (IQR).

Abbreviations: AIH, autoimmune hepatitis; ALD, alcohol‐related liver disease; BMI, body mass index; CRP, C‐reactive protein; HPN, home parenteral nutrition; INR, international normalized ratio; MASLD, metabolic dysfunction–associated liver disease; MELD, Model for End‐Stage Liver Disease; MetALD, metabolic dysfunction– and alcohol‐related liver disease; NSBB, nonselective beta‐blocker; PBC, primary biliary cholangitis; PPI, proton pump inhibitor; PSC, primary sclerosing cholangitis; SOC, standard of care. Tsd, one thousand.

^a^ Mantel‐Haenszel test was used for comparison of categorical variables.

^b^ Friedman test was used for continuous parameters.

### Statistics

We used SPSS Statistics (version 28; IBM) for the analysis of baseline characteristics. Before matching, categorial variables (number and percentage) were tested for significance using chi‐square, and continuous parameters (median and IQR) were tested with Mann‐Whitney *U* test. After 1:1 and 1:2 matching, McNemar and Mantel‐Haenszel tests were performed for the comparison of categorial variables, respectively. For continuous values, Wilcoxon and Friedman tests were used. Significance testing was performed with the use of a two‐sided alpha level of 0.05. Matching was performed with R Statistical Software (version 4.2.0; R Foundation for Statistical Computing), using RStudio and the “MatchIt” package. R Commander with plugin “EZR” was used for competing risk analyses. Given the number of different complications that can occur in patients with advanced cirrhosis, an explorative analysis without a rank‐based gatekeeping approach or a formal adjustment for multiple testing was performed.

### Ethics

Our study was approved by the Ethics Committee of Hannover Medical School (Nr 6241; German Clinical Trial Register DRKS00014615), adhering to the Declaration of Helsinki principles. Written informed consent was obtained from patients or their authorized representatives.

## RESULTS

### Clinical characteristics

#### Cohort 1

Cohort 1 (*n* = 50) had a median baseline MELD of 18 (13–22) points and a median age of 55.2 (50.1–62.4) years. The matched standard‐of‐care group (*n* = 50) was characterized by a median MELD of 17 (13–22; *P* = 0.63) and a median age of 58.7 years (50.3–62.8; *P* = 0.33). In cohort 1 and the standard‐of‐care group, 60.0% (*n* = 30) and 50.0% (*n* = 25) were male patients, respectively (*P* = 0.38). Main cause of cirrhosis was alcohol‐related in both groups (36.0% and 54.0%; *P* = 0.12). Median BMI at baseline was 20.9 (18.3–23.8) kg/$m^{2}$ in cohort 1 and 20.2 (17.8–23.3) kg/$m^{2}$ in the standard‐of‐care group (*P* = 0.31; Table 2). Most patients of cohort 1 presented with varices (*n* = 41; 82.0%) and with ascites (*n* = 40; 80.0%).

#### Cohort 2

Regarding cohort 2 and the matched standard‐of‐care group, a median baseline MELD of 16 was observed in both groups (*P* = 0.80). Furthermore, median age was 53.8 (48.0–65.5) and 57.9 (46.4–64.2) years (*P* = 0.80), and median BMI was 20.5 (17.6–23.2) kg/$m^{2}$ and 19.5 (17.3–23.4) kg/$m^{2}$ (*P* = 0.99), respectively. In both groups, cirrhosis etiology was predominantly alcohol‐related (37.5% and 53.1%, *P* = 0.22; Table 3). Main reasons against nasogastric tube feeding and percutaneous endoscopic gastrostomy tube feeding in cohort 2 were varices and ascites, as this was present in 84.4% (*n* = 27) and 81.3% (*n* = 26) of patients, respectively.

#### Application and acceptance of home parenteral nutrition

For all 50 patients who were included in cohort 2, the indication for supportive home parenteral nutrition was provided. However, only 32 patients agreed to onset of home parenteral nutrition. Reasons for declining were the invasive character of the treatment or the dependence on home care service and daily infusion regimens. Once parenteral nutrition was started, the acceptance was high, and none of the patients decided to terminate the treatment.

Most common central venous access was tunneled catheters (*n* = 13; 40.6%) at baseline, followed by nontunneled central venous catheters (*n* = 11; 34.4%) and port devices (*n* = 8; 25.0%). Most patients received standardized parenteral solutions (*n* = 28; 87.5%), with a median content of 110.0 (110.0–120.0) g carbohydrate, 56.5 (38.0–56.9) g amino acids, and 40.0 (40.0–40.0) g lipid per 1000 ml, respectively. Median daily dose was 1250.0 (1000.0–1268.8) ml. Individual compounded formulas were only administered to a minor proportion at baseline (*n* = 4; 12.5%), with a median daily dose of 1730.0 (1360.0–1886.3) ml. Median daily energy and protein intake per body weight was 33.5 (26.6–38.8) kcal/kg and 1.5 (1.2–1.8) g/kg, respectively (Table 1). Individualized compositions of parenteral nutrition contained a median of 110.0 (107.5–113.7) g carbohydrate, 50.0 (48.8–52.5) g amino acids, and 42.3 (38.3–45.0) g lipid per 1000 ml. This reflects a median daily energy and protein intake per body weight of 28.25 (26–30) kcal/kg and 1.35 (1.2–1.4) g/kg. None of the patients received primary prophylaxis for bloodstream infections. Removal of catheters was necessary in 18.8% (*n* = 6) of the patients within a 90‐day follow‐up, predominantly because of suspected or confirmed infections of the respective devices (*n* = 5; 83.3%).

### Impact of nutrition support on mortality

#### Cohort 1

Regarding all patients of cohort 1 and their matched patients, 18.0% (*n* = 9) of each group died within 90 days of follow‐up and 12.0% (*n* = 6) of cohort 1, but none of the standard‐of‐care group underwent liver transplant.

When investigating the impact of specialized nutrition support on the primary end point, no differences in mortality were detected between groups (hazard ratio [HR] = 1.06; 95% CI, 0.43–2.64; *P* = 0.90).

#### Cohort 2

Focusing on those patients who received home parenteral nutrition and their matched patients receiving standard of care, 15.6% (*n* = 15) of the patients died, and 5.2% (*n* = 5) underwent transplant. Regarding the primary end point, mortality was comparable between groups (HR = 1.44; 95% CI, 0.52–4.04; *P* = 0.48) (Table 4, Figure S4A).

**Table 4:** Clinical outcome of patients receiving SNS and of patients receiving HPN and the respective matched patients of the SOC group during 90 days of follow‐up.

Events of interest within 90 days of follow‐up	All patients (*n* = 100)	Cohort 1 (SNS group; *n* = 50)	Matched SOC group (*n* = 50)	Hazard ratio^a^	Lower 95% CI	Upper 95% CI	*P* value
Death	18 (18.0)	9 (18.0)	9 (18.0)	1.1	0.4	2.6	0.90^a^
Infection	56 (56.0)	29 (58.0)	27 (54.0)	1.2	0.7	1.9	0.60^a^
oHE							
Univariate	16 (16.0)	7 (14.0)	9 (18.0)	0.8	0.3	2.1	0.60^a^
Multivariable				0.4	0.1	1.1	0.10^a^
Nonelective readmission	32 (32.0)	15 (30.0)	17 (34.0)	0.9	0.5	1.8	0.80^a^

Events of interest within 90 days of follow‐up	All patients (*n* = 96)	Cohort 2 (HPN group; *n* = 32)	Matched SOC group (*n* = 64)	Hazard ratio^a^	Lower 95% CI	Upper 95% CI	*P* value
Death	15 (15.6)	6 (18.8)	9 (14.1)	1.4	0.5	4.0	0.48^a^
Infection	61 (63.5)	19 (59.4)	42 (65.6)	0.8	0.5	1.4	0.45^a^
Bloodstream infection	10 (10.4)	9 (28.1)	1 (1.6)	21.3	2.7	170.8	<0.01^a^
oHE							
Univariate	20 (20.8)	4 (12.5)	16 (25.0)	0.5	0.2	1.5	0.22^a^
Multivariable				0.3	0.1	0.9	0.03^a^
Nonelective readmission	33 (34.4)	12 (37.5)	21 (32.8)	1.3	0.7	2.5	0.47^a^

*Note*: Values are *n* (%).

Abbreviations: HPN, home parenteral nutrition; oHE, overt hepatic encephalopathy; SNS, specialized nutrition support; SOC, standard of care.

^a^ Results of univariate competing risk analyses.

### Impact of nutrition support on cirrhosis‐related complications

#### Cohort 1

Any infection was detected in 58.0% (*n* = 29) of cohort 1 and 54.0% (*n* = 27) of the standard‐of‐care group. Overt hepatic encephalopathy occurred in 14.0% (*n* = 7) and 18.0% (*n* = 9), respectively. In addition, about a third of the patients were nonelectively readmitted (cohort 1, 30.0%, *n* = 15; standard‐of‐care group: 34.0%, *n* = 17; Table 4). Thirty‐four percent (*n* = 34) were lost to follow‐up within the observational period.

No differences regarding the risk for nonelective hospital readmission (HR = 0.92; 95% CI, 0.47–1.80; *P* = 0.80) were detected between groups. In the standard‐of‐care group, ascites was the predominating cause for readmission (*n* = 11; 64.7%), followed by overt hepatic encephalopathy and gastrointestinal bleeding (*n* = 6; 35.3%, respectively). Likewise, the risk for overt hepatic encephalopathy (univariate: HR = 0.78; 95% CI, 0.30–2.06; *P* = 0.62; multivariable: HR = 0.38; 95% CI, 0.13–1.08; *P* = 0.07) and infections (HR = 1.16; 95% CI, 0.70–1.94; *P* = 0.57) was not ameliorated in cohort 1 (Table 4, Table S1A, Figure S3).

#### Cohort 2

In cohort 2 and the matched standard‐of‐care group, 20.8% (*n* = 20) were affected by an episode of overt hepatic encephalopathy. Rehospitalization was necessary in 34.4% (*n* = 33). Any bacterial infection was detected in almost two‐thirds (63.5%; *n* = 61) of the patients, whereas bloodstream infections occurred in 10.4% (*n* = 10) (Table 4). Additionally, 38.5% (*n* = 37) were lost to follow‐up within 90 days.

**Figure 2:**
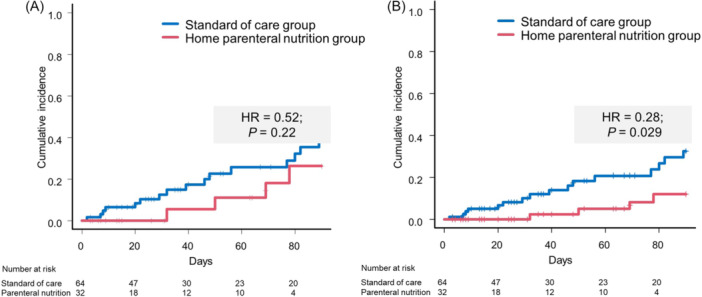
Incidence of overt hepatic encephalopathy univariate (A) and in the multivariable competing risk model (B) in the home parenteral nutrition group and the control group during 90 days of follow‐up. Hazard ratios (HRs) and *P* values report the results of competing risk analyses.

The incidence of overt hepatic encephalopathy was nonsignificantly lower in cohort 2 compared with the standard‐of‐care group (*n* = 4; 12.5% vs *n* = 16; 25.0%) (Table 4; Figure 2A). These numerical differences were not significant in the univariate analysis (HR = 0.52; 95% CI, 0.18–1.47; *P* = 0.22). Of note, a statistically significant amelioration of the risk for overt hepatic encephalopathy was detected after adjusting for rifaximin in the multivariable model (HR = 0.28; 95% CI, 0.09–0.88; *P* = 0.03) (Figure 2B). Contrastingly, no differences regarding the incidences of nonelective rehospitalization were detected (HR = 1.28; 95% CI, 0.66–2.46; *P* = 0.47) (Table 4, Figure S4B).

#### Safety of home parenteral nutrition

**Figure 3:**
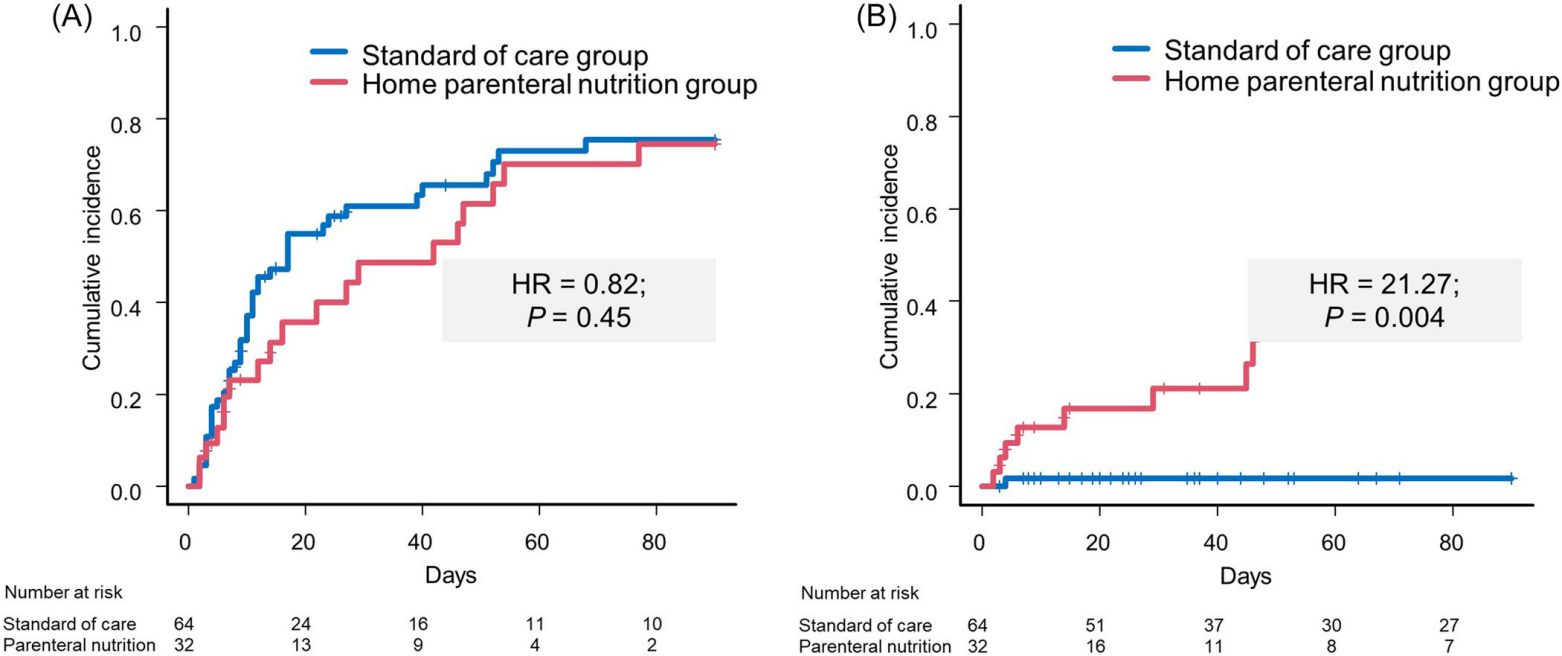
Incidence of overall bacterial infections (A) and bloodstream infections in the home parenteral nutrition group and the control group during 90 days of follow‐up. Hazard ratios (HRs) and *P* values report the results of competing risk analyses.

The overall incidence of bacterial infections was not elevated in the home parenteral nutrition group (*n* = 19; 59.4% vs *n* = 42; 65.6%), and the likelihood for bacterial infections was not aggravated in these patients (HR = 0.82; 95% CI, 0.50–1.37; *P* = 0.45) (Table 4; Figure 3A). Regarding bloodstream infections, the incidence in cohort 2 was elevated, compared with the standard‐of‐care group (*n* = 9; 28.1% vs *n* = 1; 1.6%). In line, the risk for bloodstream infections was significantly increased in the competing risk analysis (HR = 21.27; 95% CI, 2.65–170.8; *P* < 0.01) (Table 4; Figure 3B). The types of infections were dominated by spontaneous bacterial peritonitis (*n* = 19; 45.2%) and urinary tract infections (*n* = 7, 16.7%) in the standard‐of‐care group. In cohort 2, most common infection types were spontaneous bacterial peritonitis (*n* = 13; *P* = 68.4), followed by bloodstream infections (*n* = 6; 31.6%, Table S1B).

Among the different types of central venous catheters, the proportion of patients who developed a bloodstream infection or died/underwent liver transplant within 90 days was lowest in patients with ports (*n* = 1; 12.5%) and higher in those with nontunneled (*n* = 6; 54.5%) and tunneled (*n* = 8; 61.5%) catheters (*P* = 0.08).

## DISCUSSION

Targeting the nutrition status is a challenging approach in malnourished patients with liver cirrhosis. However, the impact of home parenteral nutrition on relevant cirrhosis‐specific complications remains insufficiently investigated; in particular, data about its safety in patients with advanced liver disease are scarce. In this study, parenteral feeding was associated with an increased risk for bloodstream infections but did not aggravate the overall risk for bacterial infections. Importantly, we further detected lower incidences of overt hepatic encephalopathy among patients who received home nutrition parenteral care.

In the setting of end‐stage liver diseases, malnutrition is a highly prevalent problem that affects more than half of liver transplant candidates.^2^ Underlying pathomechanisms are reduced oral dietary intake, impaired intestinal absorption, and a higher resting energy expenditure.^23^, ^24^ Among patients with decompensated cirrhosis, morbidity and mortality are even increased in the presence of malnourishment.^25^ Furthermore, poor physical conditions have been linked to higher waitlist mortality of liver transplant candidates and an elevated likelihood for postoperative complications.^26^ Therefore, several therapies addressing the nutrition status have been established. Predominantly, these strategies focus on oral diet—for example, the intake of an energy‐ and protein‐enriched diet and a higher frequency of meals to prevent fasting periods.^4^, ^27^ However, many patients are unable to meet their protein and energy demand through oral food intake. Even in a study with long‐term oral dietary intervention, nutrition was still insufficient in most patients.^6^ In line with this, specialized nutrition support, including those patients who refused parenteral nutrition and only underwent dietary counseling, did not achieve measurable impact on the clinical outcome in our study. Presumably, these patients were unable to reach an adequate oral protein and energy intake despite intensified dietary counseling.

For severely malnourished end‐stage liver disease patients, feeding via nasogastric or percutaneous endoscopic gastrostomy tubes can be considered for further enteral nutrition support.^5^ Because of esophageal varices, ascites, and severe coagulation disorders, these tubes often cannot be offered to patients with decompensated cirrhosis, and parenteral nutrition is recommended.^4^, ^28^ In these patients, parenteral nutrition is recommended by several guidelines, such as the “ESPEN guideline on clinical nutrition in liver disease.”^4^, ^5^ However, our study demonstrated a relatively high number of patients refused home parenteral nutrition, with various reasons for declining. Some patients refused parenteral nutrition because of anxiety about the invasive character of the procedure or were unwilling to be dependent on home care service and daily infusion regimens. Some patients may not be fully aware of the effects of malnutrition and the urgency of nutrition support. Nevertheless, once initiated, acceptance was high, and none of the patients decided to discontinue the treatment. The overall positive results in this study might be helpful for further acceptance of home parenteral nutrition.

Concerning cirrhosis‐specific complications, our data indicated that parenteral nutrition was associated with a significant reduction of overt hepatic encephalopathy incidences. Previous studies revealed the relevance of regular meals and adequate protein supply for encephalopathy prevention.^10^, ^29^, ^30^ Furthermore, a recent study reported an amelioration of minimal hepatic encephalopathy after repeated intravenous albumin application.^31^ When patients with liver cirrhosis undergo prolonged starving periods, catabolic pathways are activated, leading to amino acid degradation, which results in elevated circulating levels of ammonia.^11^, ^32^ Parenteral treatment avoids extended fasting periods and might therefore alleviate the imbalance between catabolic and anabolic processes. Despite the relatively low number of patients as one limiting factor of our study, the beneficial effect of home parenteral nutrition treatment reached statistical significance. In consequence, the application of parenteral nutrition as a therapeutic tool for the prevention of hepatic encephalopathy needs to be further evaluated.

Importantly, decompensated cirrhosis is characterized by a high infection vulnerability that might affect safety of permanent central venous catheter insertion.^16^ Infections are life‐threatening events, often promoting further decompensation, acute‐on‐chronic liver failure, and death.^33^, ^34^, ^35^ Causal mechanisms of the acquired immunodeficiency in cirrhosis are alterations of the innate and adaptive immunity, known as cirrhosis‐associated immune dysfunction.^36^ More than half of our overall cohort acquired any infection during follow‐up. As the implantation of a permanent central venous catheter allows direct access to patients’ blood system, it might be the primary vulnerability of parenteral treatment. In accordance with this hypothesis, our data revealed an increased risk for bloodstream infections in patients receiving parenteral nutrition. This finding was in accordance with several other studies that reported a linkage between bloodstream infections and insertion of permanent catheters.^37^, ^38^, ^39^ However, the likelihood for overall infection acquirement, as well as the risk for nonelective readmission and mortality, was not elevated in the parenteral group in our study. Presumably because of the high incidence of overall infections also in the standard‐of‐care group and because some patients of the parenteral nutrition cohort already developed bloodstream infections during their baseline hospital stay, readmission rates were comparable between groups. Furthermore, increased mortality rates might have been prevented because of close monitoring of patients treated parenterally, prompt treatment, and therefore, high controllability of occurring bloodstream infections. Hence, attending physicians should be aware of the higher likelihood for bloodstream infections and ensure intensive monitoring of these patients. Furthermore, the increased incidence of bloodstream infections in the parenteral nutrition group could have been a trigger for overt hepatic encephalopathy development, which was not observed in our study. This further strengthens the positive effect of home parenteral nutrition on the risk for hepatic encephalopathy. Consequently, our data suggest that the linkage between central lines and bloodstream infections should not be treated as a prohibiting factor for the onset of home parenteral nutrition in severely malnourished patients awaiting liver transplant. As a limitation, routine antibiotic prophylaxis, as recommended in current guidelines,^40^ was not part of standard care during the study period; however, to minimize risk of infection, all patients received catheter care according to established hygienic standards at the time of study initiation. Regarding further limitations, the patients who refused parenteral nutrition and only received repetitive dietary counseling have to be mentioned, as adequate nutrition support could not be guaranteed in these cases. As this group was larger than initially expected, we decided on separate analyses of patients with parenteral nutrition and patients with overall nutrition support. Nevertheless, these problems are also characteristic for clinical routine, which might be explained by the shortage of current evidence for the benefits of home parenteral feeding. Standard‐of‐care treatment that was received by the controls only included a nonstandardized basic nutrition counseling, which might have not been adequate to reach individual energy goals. Another common limitation is the detection of malnutrition. Nutrition status was assessed using the NRS‐2002, a validated screening tool for risk of malnutrition recommended by ESPEN. Although liver disease–specific tools such as the Royal Free Hospital Nutritional Prioritizing Tool may be more sensitive in this population, their use was not feasible because of the retrospective design and the timing of cohort initiation. Furthermore, BMI and bioelectrical impedance analyses may be confounded by the presence of ascites or anasarca in patients with decompensated cirrhosis. Therefore, these results were interpreted cautiously, with acknowledgment that ascites and fluid shifts may confound impedance‐based measurements.

Furthermore, the relatively high number of patients lost to follow‐up and the lack of a randomized controlled study design, which necessitated matching between patients with and without nutrition support, reveal an additional limitation. Although propensity score matching mitigates confounding for the matched covariates, we cannot exclude that other parameters, potentially influencing the likelihood for certain outcomes, might differ between the compared groups. When interpreting the results of the secondary analyses, the missing adjustment for multiple testing must be taken into account also. However, anything but an explorative analysis would not be possible, considering the number of different complications that can occur in this highly vulnerable population. Hence, randomized controlled studies would be worthwhile to confirm the results of our analysis.

In addition to nutrition support, a tailored approach to physical activity represents an important component in the management of malnutrition, sarcopenia, and frailty. Exercise interventions adapted to individual functional capacity may help preserve muscle mass, improve physical performance, and potentially enhance the effectiveness of nutrition therapy.^41^ Future prospective studies should therefore focus on combined nutrition and exercise‐based interventions to better define their synergistic effects on clinical and functional outcomes.

In conclusion, home parenteral nutrition does not seem to be contraindicated in this highly vulnerable collective, strengthening the evidence for current guideline recommendations. Additionally, the reduced likelihood for overt hepatic encephalopathy suggests promising effects of home parenteral nutrition with regard to this relevant cirrhosis‐specific complication. Further studies are needed to corroborate these findings in enlarged cohorts and thereby provide further evidence for the clinical management of severely malnourished patients with end‐stage liver disease.

## AUTHOR CONTRIBUTIONS

**Katharina L. Hupa‐Breier:** Conceptualization; writing – review and editing; and supervision. **Laura Buttler:** Conceptualization; data assessment; methodology; investigation; formal analysis; and writing – original draft. **Claudia Seipt:** Data assessment and writing – review and editing. **Birgit Kaufmann:** Data assessment and writing – review and editing. **Marie Griemsmann:** Data assessment and writing – review and editing. **Tammo Tergast:** Data assessment and writing – review and editing. **Andrea Markowski:** Data assessment and writing – review and editing. **Hannah Rieland:** Formal analysis and writing – review and editing. **Heiner Wedemeyer:** Conceptualization and writing – review and editing. **Benjamin Maasoumy:** Conceptualization; methodology; writing – review and editing; and supervision. **Andrea Schneider:** Conceptualization; methodology; data assessment; and writing – review and editing.

## CONFLICT OF INTEREST STATEMENT

Katharina L. Hupa‐Breier received a lecture fee from Falk Pharma, Eli Lilly, and NovoNordisk. Heiner Wedemeyer served as a speaker and/or advisory board member for Abbott; Biotest AG; Bristol‐Myers‐Squibb; Hoffmann/La Roche Ltd; GlaxoSmithKline Services Unlimited; Janssen; Roche Diagnostics; Vir Biotechnology, Inc; and Gilead Science and received research support from Abbott and Biotest AG. Benjamin Maasoumy served as a speaker and/or advisory board member for AbbVie, Fujirebio, Gilead, Luvos, MSD, Norgine, Roche, and W. L. Gore & Associates and received research support from Altona, EWIMED, Fujirebio, and Roche. The remaining authors declare no conflicts of interest.

## Supporting information

Supplementary figure 1: Love plot (A) and jitter plot (B) of patients receiving specialized nutrition support (SNS) and their matched controls. Abbreviations: BMI, body mass index; MELD, Model for End‐Stage Liver Disease.

Supplementary figure 2: Love plot (A) and jitter plot (B) of patients receiving parenteral nutrition (HPN) and their matched controls. Abbreviations: BMI, body mass index; MELD, Model for End‐Stage Liver Disease.

Supplementary figure 3: Mortality (A), incidence of nonelective rehospitalization (B), bacterial infections (C), and oHE (D) in patients receiving specialized nutrition support (SNS) and their controls during 90 days of follow‐up. Hazard ratios (HRs) and *P* values report the results of competing risk analyses. Abbreviations: SNS, specialized nutrition support; SOC, standard of care.

Supplementary figure 4: Mortality (A) and nonelective hospital readmission (B) in the home parenteral nutrition (HPN) group and the control group during 90 days of follow‐up. Hazard ratios (HRs) and *P* values report the results of competing risk analyses. Abbreviation: SOC, standard of care.

Supplementary Table 1A: Type of infections in patients receiving specialized nutrition support (SNS) and the standard‐of‐care (SOC) group. Supplementary Table 1B: Type of infections in patients receiving parenteral nutrition (HPN) and the standard‐of‐care (SOC) group.
