# Supplementary figures and images for: Association between specialized nutrition support and 90‐day mortality relative to standard of care in malnourished adults with decompensated cirrhosis: A retrospective cohort study
Source: JPEN J Parenter Enteral Nutr. 2026 Feb 20;50(4):544–56. doi: 10.1002/jpen.70066 (PMC13169273; doi:10.1002/jpen.70066)

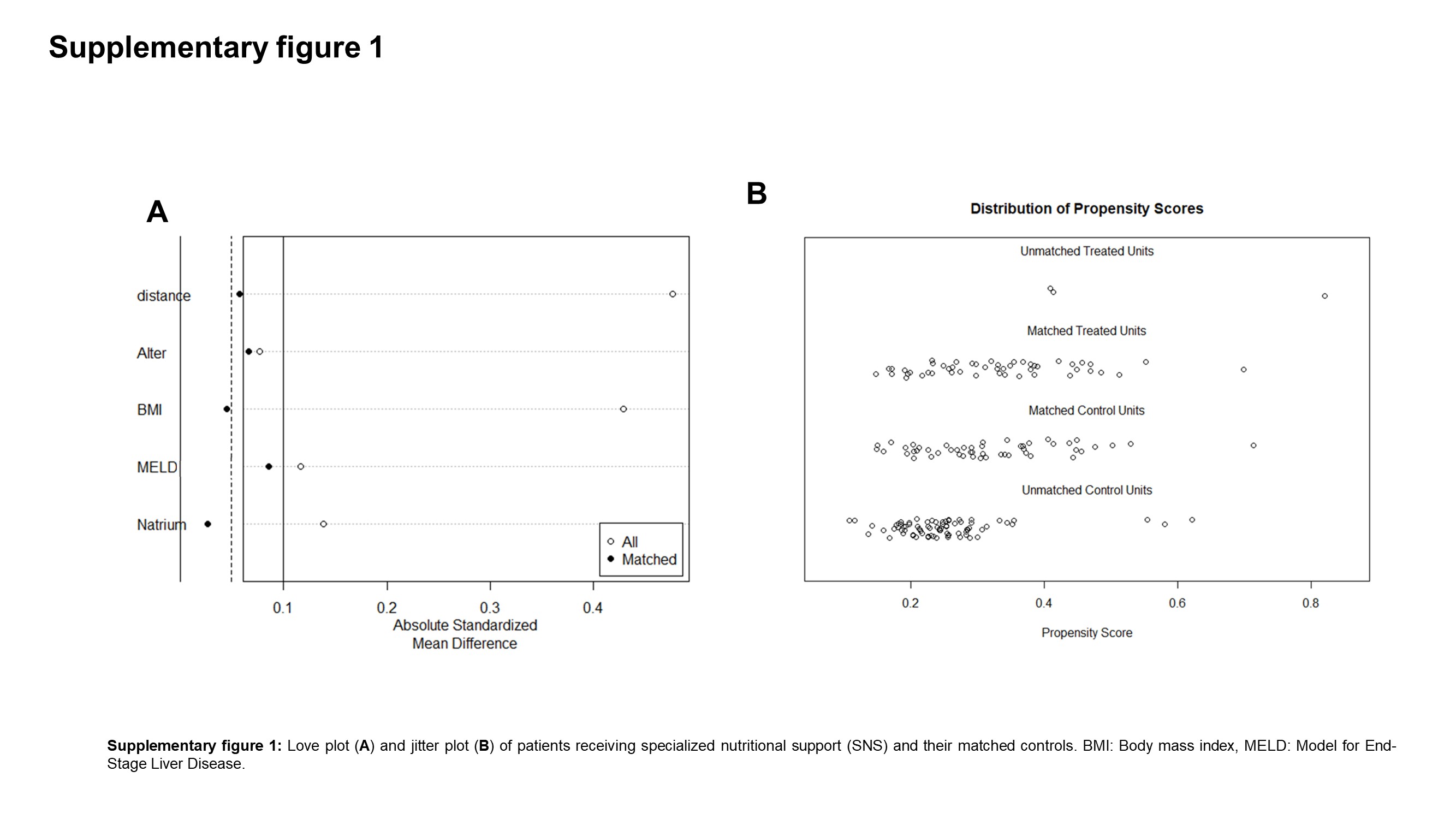

Supplement: Supplementary file 1 — Supplementary figure 1: Love plot (A) and jitter plot (B) of patients receiving specialized nutrition support (SNS) and their matched controls. Abbreviations: BMI, body mass index; MELD, Model for End‐Stage Liver Disease. [file JPEN-50-544-s005.jpg]

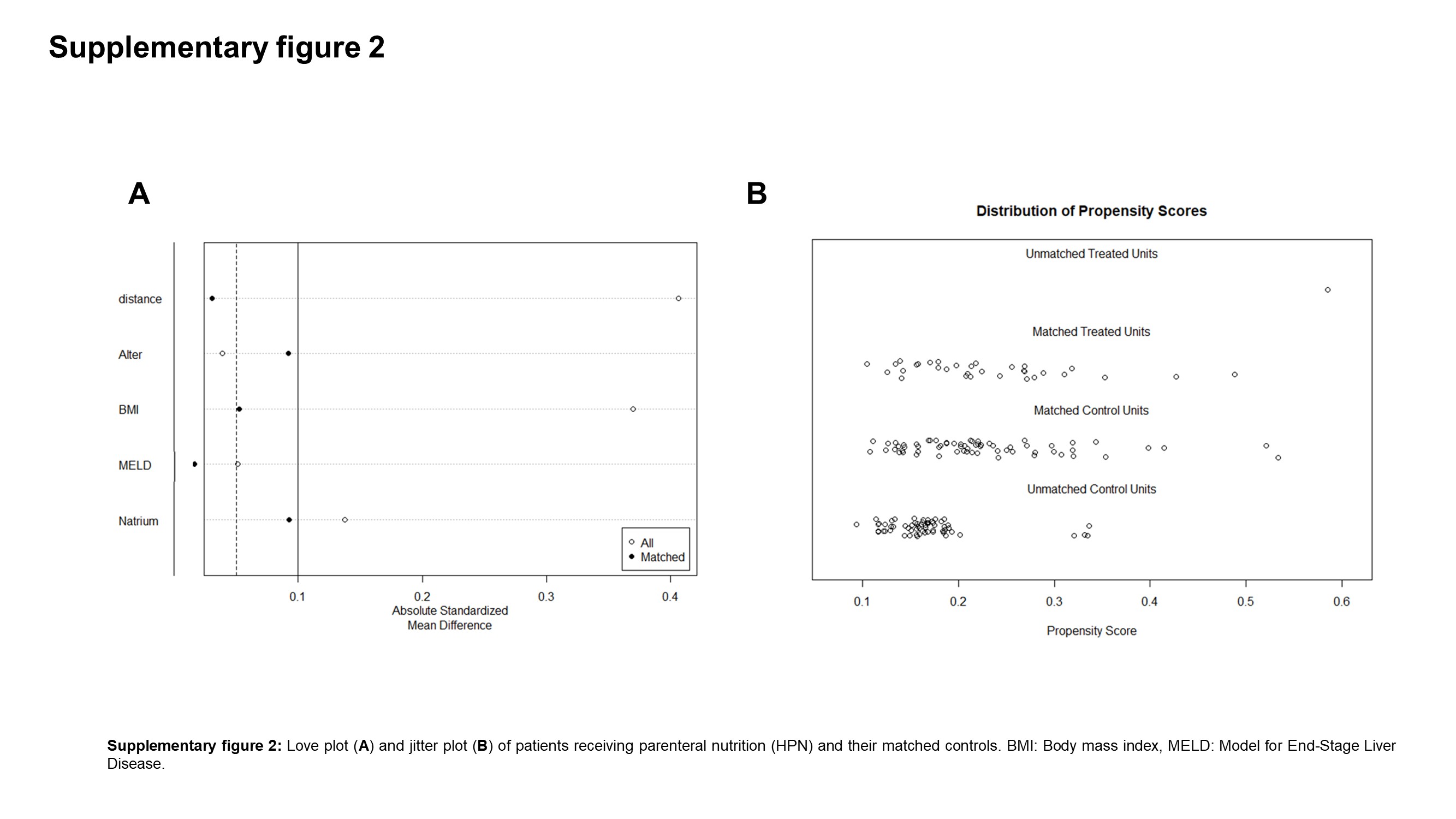

Supplement: Supplementary file 2 — Supplementary figure 2: Love plot (A) and jitter plot (B) of patients receiving parenteral nutrition (HPN) and their matched controls. Abbreviations: BMI, body mass index; MELD, Model for End‐Stage Liver Disease. [file JPEN-50-544-s001.jpg]

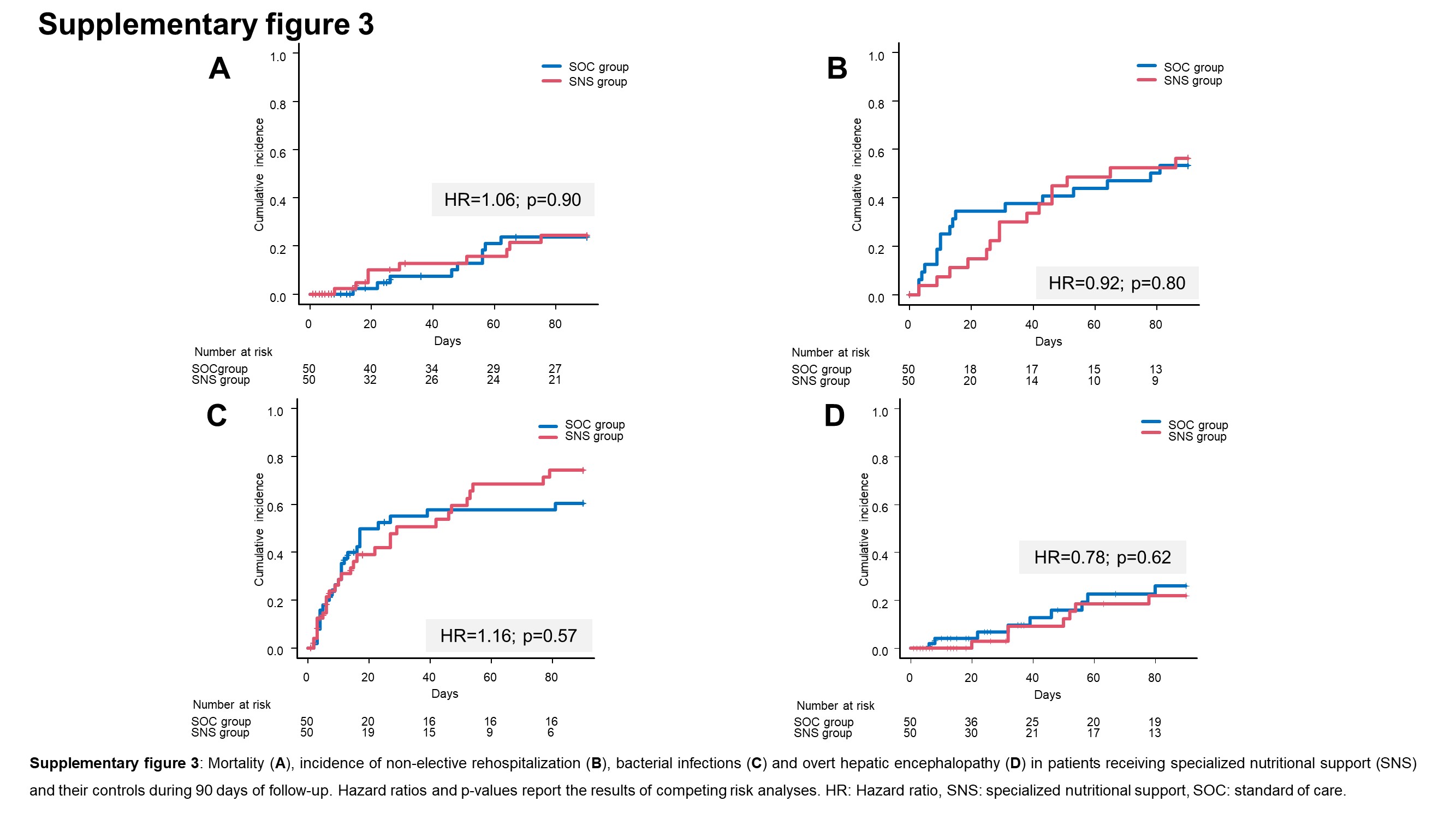

Supplement: Supplementary file 3 — Supplementary figure 3: Mortality (A), incidence of nonelective rehospitalization (B), bacterial infections (C), and oHE (D) in patients receiving specialized nutrition support (SNS) and their controls during 90 days of follow‐up. Hazard ratios (HRs) and P values report the results of competing risk analyses. Abbreviations: SNS, specialized nutrition support; SOC, standard of care. [file JPEN-50-544-s003.jpg]

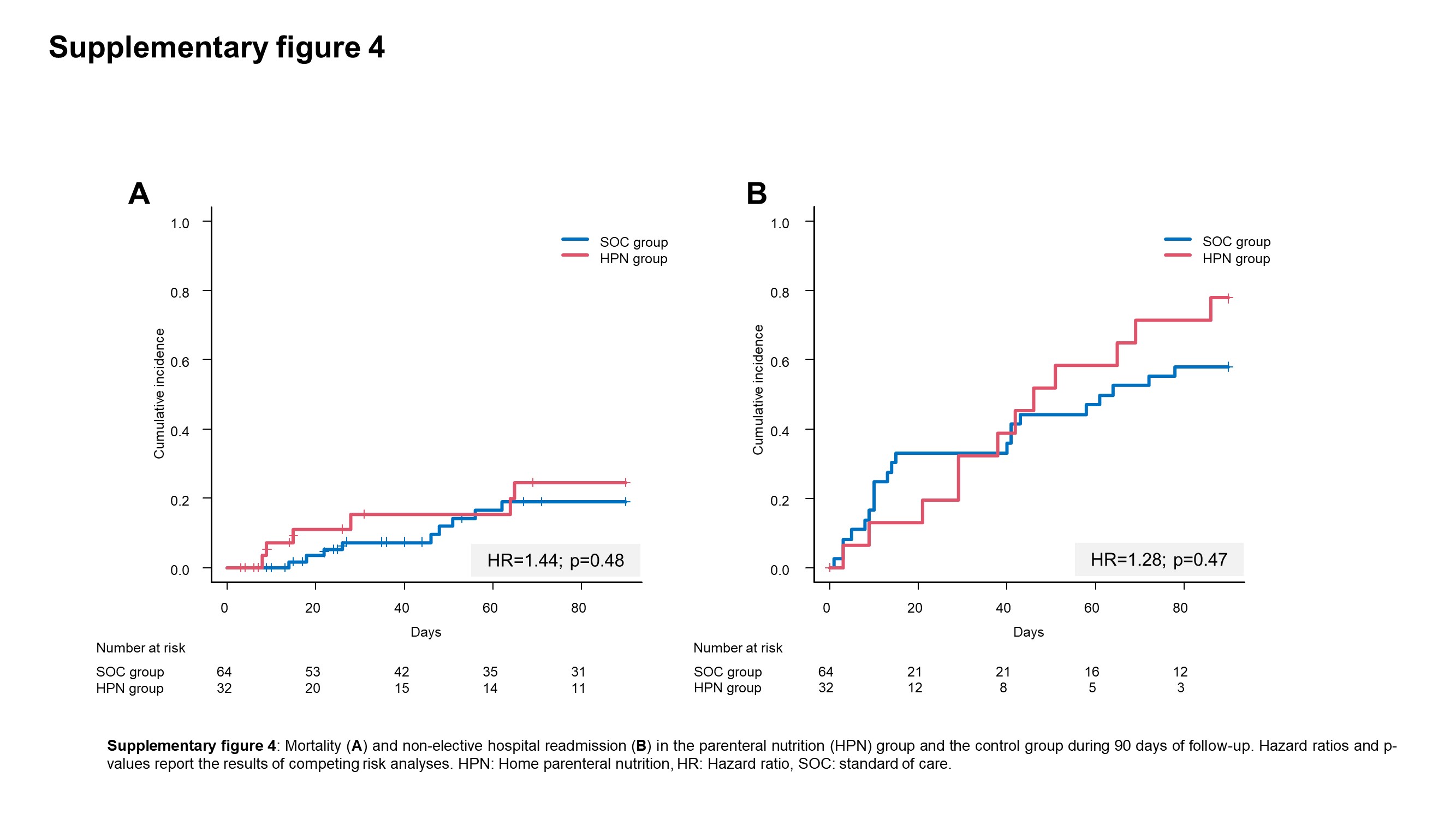

Supplement: Supplementary file 4 — Supplementary figure 4: Mortality (A) and nonelective hospital readmission (B) in the home parenteral nutrition (HPN) group and the control group during 90 days of follow‐up. Hazard ratios (HRs) and P values report the results of competing risk analyses. Abbreviation: SOC, standard of care. [file JPEN-50-544-s004.jpg]
