# Supplementary material for: Association between specialized nutrition support and 90‐day mortality relative to standard of care in malnourished adults with decompensated cirrhosis: A retrospective cohort study
Source: JPEN J Parenter Enteral Nutr. 2026 Feb 20;50(4):544–56. doi: 10.1002/jpen.70066 (PMC13169273; doi:10.1002/jpen.70066)
Supplement: Supplementary file 5 — Supplementary Table 1A: Type of infections in patients receiving specialized nutrition support (SNS) and the standard‐of‐care (SOC) group. Supplementary Table 1B: Type of infections in patients receiving parenteral nutrition (HPN) and the standard‐of‐care (SOC) group. [file JPEN-50-544-s002.docx]

**Supplementary tables**

**Supplementary table 1A:** Type of infections in patients receiving specialized nutritional support (SNS) and the standard of care (SOC) group.

| Infection type* | Cohort 1 (SNS group; n=50)  (29 patients with infection) | Matched SOC group (n=50)  (27 patients with infection) |
| --- | --- | --- |
| Spontaneous bacterial peritonitis | 15 (51.7) | 18 (66.7) |
| Urinary tract infection | 4 (13.8) | 7 (25.9) |
| Pneumonia | 1 (3.4) | 0 (0.0) |
| Bloodstream infection | 5 (17.2) | 1 (3.7) |
| Unknown source | 4 (13.8) | 2 (7.4) |
| Other | 3 (10.3) | 4 (14.8) |

Values as n (%).

*Multiple selection possible.

**Supplementary table 1B:** Type of infections in patients receiving parenteral nutrition (HPN) and the standard of care (SOC) group.

| Infection type* | Cohort 2 (HPN group; n=32)  (19 patients with infection) | Matched SOC group (n=64)  (42 patients with infection) |
| --- | --- | --- |
| Spontaneous bacterial peritonitis | 13 (68.4) | 19 (45.2) |
| Urinary tract infection | 3 (15.7) | 7 (16.7) |
| Pneumonia | 0 (0.0) | 4 (9.5) |
| Bloodstream infection | 6 (31.6) | 1 (2.4) |
| Unknown source | 2 (10.5) | 6 (14.3) |
| Other | 1 (5.3) | 7 (16.7) |

Values as n (%).

*Multiple selection possible.
